# Fusion of Facial Expressions and EEG for Multimodal Emotion Recognition

**DOI:** 10.1155/2017/2107451

**Published:** 2017-09-19

**Authors:** Yongrui Huang, Jianhao Yang, Pengkai Liao, Jiahui Pan

**Affiliations:** School of Software, South China Normal University, Guangzhou 510641, China

## Abstract

This paper proposes two multimodal fusion methods between brain and peripheral signals for emotion recognition. The input signals are electroencephalogram and facial expression. The stimuli are based on a subset of movie clips that correspond to four specific areas of valance-arousal emotional space (happiness, neutral, sadness, and fear). For facial expression detection, four basic emotion states (happiness, neutral, sadness, and fear) are detected by a neural network classifier. For EEG detection, four basic emotion states and three emotion intensity levels (strong, ordinary, and weak) are detected by two support vector machines (SVM) classifiers, respectively. Emotion recognition is based on two decision-level fusion methods of both EEG and facial expression detections by using a sum rule or a production rule. Twenty healthy subjects attended two experiments. The results show that the accuracies of two multimodal fusion detections are 81.25% and 82.75%, respectively, which are both higher than that of facial expression (74.38%) or EEG detection (66.88%). The combination of facial expressions and EEG information for emotion recognition compensates for their defects as single information sources.

## 1. Introduction

Emotion plays a powerful role in social influence: not only does it include psychological responses to external stimuli or one's own stimuli but it is also accompanied by physiological responses to psychological reactions in individuals' daily lives. Emotional influences are manifested across a variety of levels and modalities [1]. On the one hand, peripheral signals are related to the somatic nervous system and show physiological changes in emotion states. For instance, there are physical signals that emerge: facial expressions, verbal speech, or body language. On the other hand, there are also influences on cognitive processes, including coping behaviors such as wishful thinking, resignation, or blame-shifting. The goal of our research is to perform a multimodal fusion between EEGs and peripheral physiological signals for emotion recognition.

Previous studies have investigated the use of peripheral and brain signals separately, but little attention has been paid thus far to a fusion between brain and peripheral signals. In one study, Ekman and Friesen made a pioneering contribution to modern facial expression recognition [2]. They defined the six basic expressions of human beings, that is, pleasure, anger, surprise, fear, disgust, and sadness, and identified the categories of objects to be investigated. Mase made use of optical flow to determine the main direction of movement of the muscles and then constructed the Face Recognition System [3]. Picard and Daily at MIT Media Laboratory developed pattern recognition algorithms that attained 78.4% classification accuracy for three categories of emotion states using the peripheral signals of galvanic skin resistance, blood pressure, respiration, and skin temperature [4].

Compared to periphery physiological signals, EEG signals have been proven to provide greater insights into emotional processes and responses. Furthermore, because EEG has been widely used in BCIs, the study of EEG-based emotion detection may provide great value for improving the user experience and performance of BCI applications. Chanel et al. reported an average accuracy of 63% by using EEG time-frequency information as features and support vector machine (SVM) as a classifier to characterize EEG signals into three emotion states [5]. Nasehi et al. made use of quadratic discriminant analysis and SVM to classify emotions into the six categories of pleasure, surprise, anger, fear, disgust, and sadness, achieving accuracies of 62.3% and 83.33%, respectively [6]. Ishino and Hagiwara categorized user status into four emotion states using neural networks with accuracies ranging from 54.5% to 67.7% for each of the four emotion states [7]. However, the use of EEG-based emotion recognition is still in its infancy.

In recent years, with the development of multisource heterogeneous information fusion processing, it has become possible to fuse features from multicategory reference emotion states. The use of different types of signals to support each other through supplementary information fusion processing can be greatly improved. Therefore, people have begun to use facial expressions, voice messages, eye movements, gestures, and physiological signals and other channels of emotional information between the complementarity to study identification problems, that is, based on multimodal emotion recognition [8]. Most previous works have focused on the fusion of audiovisual information for automatic emotion recognition, for example, combining speech with facial expression. Busso et al. proposed a rule-based decision-level fusion method for combined analysis of speech and facial expressions [9]. Wanger et al. used boosting techniques to automatically determine adaptive weights for audio and visual features [10]. A few studies have focused on the multimodal fusion of EEG and physiological signals. In a study [11], the International Affective Picture System (IAPS) was utilized as stimuli, and the use of self-assessment labels for arousal assessment yielded accuracies of 55%, 53%, and 54% for EEG, physiological, and fused features, respectively. All of the studies have shown that the performances of emotion recognition systems can be improved by employing multimodal information fusion.

In this study, we propose two multimodal fusion methods combining brain and peripheral signals for emotion recognition. The input signals are electroencephalogram and facial expression. The stimuli are based on a subset of movie clips that correspond to four specific areas of valance-arousal emotional space (happiness, neutral, sadness, and fear). For facial expression detection, four basic emotion states are detected by a neural network classifier. For EEG detection, four basic emotion states and three emotion intensity levels (strong, ordinary, and weak) are detected by two SVM classifiers, respectively. Emotion recognition is based on two decision-level fusion methods of both EEG and facial expression detections by using a sum rule or a production rule. Twenty healthy subjects attended two experiments. The results show that the accuracies of two multimodal fusion detections are 81.25% and 82.75%, respectively, which are both higher than that of facial expression or EEG detection. The combination of facial expressions and EEG information for emotion recognition compensates for their defects as single information sources.

## 2. Methods

### 2.1. Data Acquisition System

A Mindwave Mobile device (Neurosky, Inc., Abbotsford, Australia) was used to capture scalp EEG signals, and a Logitech camera (25 FPS, 800 × 600 image size) was used to capture facial expressions. According to the standard 10–20 system, the EEG signals are referenced to the right mastoid. The EEG signals used for analysis were recorded from the “Fz” electrode. The impedances of all electrodes were maintained below 5 kΩ.

### 2.2. Data Processing and Algorithm

**Figure 1:**
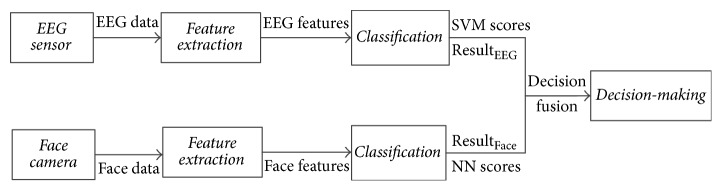
Data processing procedure of the multimodal emotion recognition.

For our proposed system, the EEG and facial expression detectors were designed separately. The EEG and image data were fed into the two detection procedures simultaneously.  Figure 1 shows the data processing procedure. The analysis methods and algorithms used in this study are described below.

#### 2.2.1. Facial Expression Detection

For the face features extraction, the face position is detected in real-time by the AdaBoost algorithm based on the Haar eigenvalue. The Haar classifier uses the AdaBoost algorithm of the Boosting algorithm, resulting in a cascade of weak classifiers trained by the AdaBoost algorithm. We use the Haar-like feature in image as input of the classifier. The output of the classifier is whether this image is human face [12]. When the human face from the input image is found, we resize it into 48-pixel width and 48-pixel height. Next, the PCA method is used to reduce the dimensionality. The output of this phrase is 169 dimensions (obtained by the grid search method mentioned below) after dimensionality reduction.

**Figure 2:**
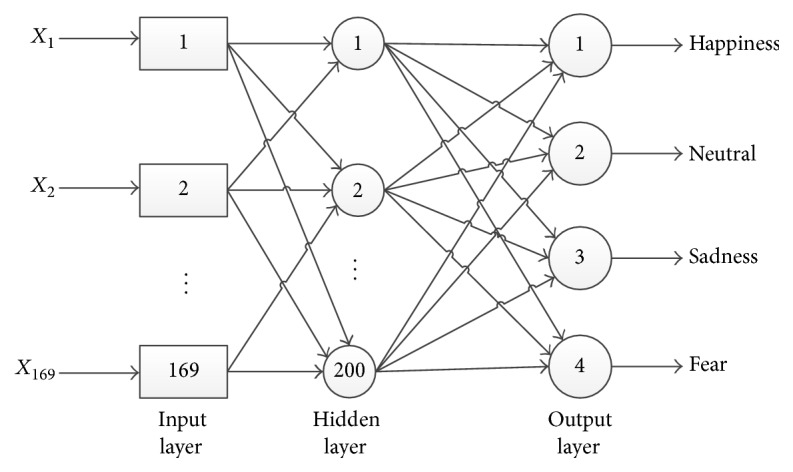
The architecture of the proposed system for face expression classification: the network has one hidden layer with 200 neurons. The input of this network is 169 image features we get from dimensionality reduction, while the output is the scores of four emotion states (happiness, neutral, sadness, and fear). The learning rate of this network is 0.1. We use sigmoid function as the activation function of this network.

The feature vectors are then fed to a feedforward neural network.  Figure 2 shows the architecture of the proposed system for face expression classification. Applying the trained feedforward neural network classifier to the face image feature, we obtain four scores (the values of the objective function of the feedforward neural network), denoted as *s*1_*j*_  (*j* = 1,…, 4). *j* represents the four emotion states (happiness, neutral, sadness, and fear) detected by face expression classifier. We normalize the four scores by mapping them to the range [0,1].

(1) s^1j=s1j−min⁡s11,…,s14max⁡s11,…,s14−min⁡s11,…,s14

(2) r1=arg⁡maxj⁡s^1j.

The four normalized scores $\hat{s}1_{j}\;(j=1,2,3,4)$ representing the output of feedforward neutral network are used in the decision-making step described later, and *r*_1_ represents the emotion state detected by face expression.

Note that the hyperparameters of the face expression classifier are determined by the grid search method, a brute-force searching through a manually specified subset of the hyperparameters space of a learning algorithm. We initialize the subset of the hyperparameters space in

(3) D∈121,144,169,225N∈150,200,250,300R∈0.001,0.01,0.1,1,

where *D* is the number of the dimensions, *N* is the number of the neurons in hidden layer, and *R* is the learning rate in classification. Grid search then trains a classifier with each pair (*D*, *N*, *R*) in the Cartesian product of these three sets and evaluates their performance on a held-out validation set. We select the classifier parameters with the best performance and apply it into our model.

#### 2.2.2. EEG Detection

The EEG-based detection includes two progressive stages: feature extraction based on PSD and classification using SVM. The analysis methods and algorithms used in this study are described below.

The EEG data are bandpass filtered over eight frequency bands: delta (1–3 Hz); theta (4–7 Hz); alpha1 (8–10 Hz); alpha2 (11–13 Hz); beta1 (14–20 Hz); beta2 (21–30 Hz); gamma1 (31–40 Hz); and gamma2 (41–50 Hz). We compute the traditional PSD features using the Short Time Fourier Transform (STFT) with a 1-s window and no overlapping Hanning window. For classification, we use two linear SVM classifiers here, one for the emotion states classification, and one for the emotion intensities classification. We train samples (*x*_*i*_, *y*_*i*_) and (*x*_*i*_, *y*_*i*_′),

(4) xi=DELTA,THETA,ALPHA1,ALPHA2,BETA1,BETA2,GAMMA1,GAMMA2

(5) yi=1happiness2neutral3sadness4fear

(6) yi′=−1weak0moderate1strong,

where DELTA, THETA, ALPHA1, ALPHA2, BETA1, BETA2, GAMMA1, and GAMMA2 represent the power density spectrum corresponding to the eight frequency bands mentioned above, *y*_*i*_ represents the label of the four emotion states, *y*_*i*_′ is the label of the three emotion intensity levels, and *x*_*i*_ represents the feature vectors corresponding to the four emotion states or the three emotion intensity levels.

Applying the first trained SVM classifier to the feature vectors, we obtain four scores (the values of the objective function of the SVM), denoted as *s*2_*j*_  (*j* = 1,…, 4). *j* represents the four emotion states (happiness, neutral, sadness, and fear) detected by EEG classifier. We normalize the four scores by mapping them to the range [0,1].

(7) s^2j=s2j−min⁡s21,…,s24max⁡s21,…,s24−min⁡s21,…,s24r2=argmaxj⁡s^2j.

The four normalized scores $\hat{s}2_{j}\;(j=1,2,3,4)$ and the index of the maximum score *r*_2_ representing the output of the emotion state in the EEG detection are used in the first fusion method described later.

Applying the second trained SVM classifier to the feature vectors, we obtain three scores *s*2_*k*_′  (*k* = 1,2, 3) corresponding to the three emotion intensity levels (weak, moderate, and strong), and find the index of the maximum score.

(8) $$\begin{array}{c} r_{2}^{\prime }=\underset{k}{\arg\max}\left(\;s2_{k}^{\prime }\;\right). \end{array}$$

The index of the maximum score *r*_2_′ representing the output of the emotion intensity level in the EEG detection is used in the second fusion method described later.

#### 2.2.3. Classification Fusion

In the decision-level fusion, the outputs generated by two classifiers of the facial expression and EEG detections are combined. We employ two fusion methods of both EEG and facial expression detections as follows.

For the first fusion method, we have applied the sum strategy (e.g., [12]) to the decision-level fusion. Specifically, we calculate the sum of the normalized face expression classifier scores $\hat{s}1$ and EEG classifier scores $\hat{s}2$ for each of the four emotion states. Finally, we find the maximum of the four summed values as shown as follows:

(9) sumj=s^1j+s^2jj=1,2,3,4rsum=argmaxj⁡sumj,

where $\hat{s}1_{j}\;(j=1,2,3,4)$ and $\hat{s}2_{j}\;\;(j=1,2,3,4)$ are calculated in (1) and (6), and *r*_sum_ is the index corresponding to the maximum of the summed values.

For the second fusion method, we adopt the decision-making strategy based on production rules, which are commonly used as a simple expert system in the cognitive modeling and artificial intelligence (e.g., [13, 14]). Through the production rule, the four emotion states (happiness, neutral, sadness, or fear) and the three emotion intensity levels (strong, moderate, or weak) are combined to emotion recognition. A production rule consists of an IF part (a condition or premise) and a THEN part (an action or conclusion). The form of production rules is

(10) $$\begin{array}{c} Ri:IF\;P\;THEN\;Q, \end{array}$$

where *Ri* represents the rule *i*, *P* is the antecedent of rule *i*, and *Q* is the latter of rule *i*. In this study, *P* is formed by (*r*_1_, *r*_2_′). *r*_1_ represents the emotion state detected by facial expression, while *r*_2_′ represents the emotion intensity level detected by EEG. The production rules are defined as shown in Table 1.

All the rules will be triggered as soon as their conditions are met. For example, in the production rule *R*_3_, if the emotion state detected by facial expression is happiness and the emotion intensity level detected by EEG is weak, the final result of the emotion recognition is neutral.

## 3. Experiment

Two experiments, offline and online, were conducted in this study. In this study, the data of the first experiment was used for training. Twenty healthy 19- to 33-year-old subjects from the local research unit attended the experiments. During the experiments, the subjects were seated in a comfortable chair and instructed to avoid blinking or moving their bodies.

### 3.1. Experiment 1 (Offline)

The data collected in this experiment consisted of 40 trials for each subject. At the beginning of each trial, a fixation cross was first presented at the center of the GUI to capture the subjects' attention. After 2 s, movie clips inducing different emotional conditions were presented at the center of the GUI in a random order. Each movie clip was presented for 2-3 minutes, preceded by 5 s of a blank screen as the start hint. At the ends of trials, subjects were asked to view each movie clip, assign valence and arousal ratings, and rate the specific emotions they had experienced. The rating procedure lasted approximately 60 s. There was a 10-s break between two consecutive trials for emotional recovery. During each trial, we collected 100 human face images using a camera and 200 groups of EEG signals using a Mindwave Mobile device. Valence and arousal ratings were obtained using the Self-Assessment Manikin (SAM) [15]. Four basic emotion states (happiness, neutrality, sadness, and fear) and three emotion intensities (strong, ordinary, and weak) were evaluated in this study. The given self-reported emotion states and intensity level were used to verify the facial and EEG emotion classifications. We used images and corresponding emotion states to train a feedforward neural network classifier. We used EEG signals and corresponding emotion states to train a SVM classifier. A different neural network classifier and a different SVM classifier were fitted to each subject. They were both used in Experiment 2 to detect emotion states.

### 3.2. Experiment 2 (Online)

**Figure 3:**
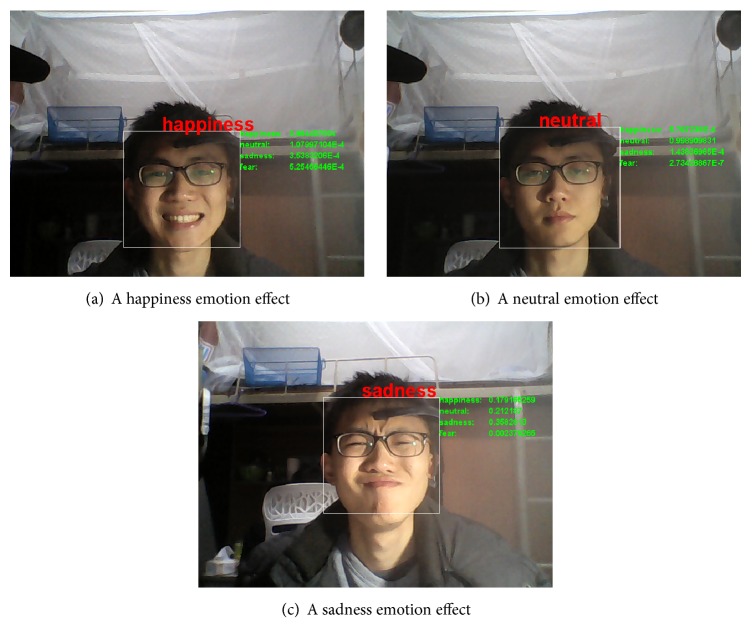
Example screenshots of face videos from Experiment 2.

This experiment was composed of 40 trials for each subject, corresponding to the 40 movie clips evaluated in Experiment 1. The procedure of each trial was similar to that in Experiment 1. However, at the end of each movie clip, 3 different detectors (a face expression detector, EEG detectors, and the first fusion detector) were used to determine the emotion state. If the detection result was correct, positive feedback consisting of auditory applause occurred for 4 s. Otherwise, no feedback was given. For performance evaluation, the online accuracy was calculated as the ratio of the number of correct predictions to the total number of presented trials.  Figure 3 shows several screenshots of face videos from Experiment 2.  Figure 3(a) shows a subject who was watching a lively movie clip.  Figure 3(b) shows a subject who was watching a normal movie clip.  Figure 3(c) shows a subject who was watching a sad movie clip.

### 3.3. Data Analysis (Offline)

To validate the second fusion method combining type of emotion and intensity level, an offline data analysis was conducted. For the data set of Experiment 1, we used images and corresponding emotion states to train a feedforward neural network classifier and used EEG signals and corresponding emotion intensities to train a SVM classifier. For the data set of Experiment 2, we used the second fusion detector based on the production rules to determine the emotion state and calculated the corresponding offline accuracy rates.

## 4. Results

The average accuracies of the two fusion methods for twenty subjects are shown in Table 2. The classification accuracies of the face expression detection and the EEG detection are also shown in Table 2. It shows that the accuracy of the first fusion detection using a sum rule is 81.25% and the accuracy of the second fusion detection using a production rule is 82.75%, which are both higher than that of facial expression (74.38%) or EEG detection (66.88%). Specifically, seventeen of 20 subjects achieved the highest accuracies using the fusion methods. Moreover, accuracies in each of the three detections were tested using paired *t*-test. Results were considered significant when *p* values were below 0.05. The statistical analysis based on *t*-test indicated the following: (i) higher accuracies were achieved for the two fusion methods than for the face expression detection or the EEG detection (the first fusion method versus face expression detection, *p* = 0.03; the first fusion method versus EEG detection, *p* < 0.01; the second fusion method versus face expression detection, *p* = 0.03; the second fusion method versus EEG detection, *p* < 0.01); (ii) the accuracies were not significantly different between the face expression detection and the EEG detection (EEG detection versus face expression detection, *p* = 0.08); (iii) the accuracies were also not significantly different between the first and the second fusion methods (the first fusion method versus the second fusion method, *p* = 0.56). Furthermore, we can see that low accuracies were obtained for subjects 5, 19, and 20. That could be attributed to them having less expressive facial expressions or perhaps our approach is less sensitive to them.

## 5. Discussions

This paper employs information fusion technology combined with facial expression recognition technology and EEG emotion recognition technology. The stimuli are based on a subset of movie clips that correspond to four specific areas of valance-arousal emotional space (happiness, neutral, sadness, and fear). The four emotion states are detected by both facial expression and EEG. Emotion recognition is based on a decision-level fusion of both EEG and facial expression detection. Twenty healthy subjects attended two experiments. The results show that the accuracies of two information fusion detections are 81.25% and 82.75%, which are both higher than that of facial expression (74.38%) or EEG detection (66.88%).

The notion that combining brain and peripheral physiological signals will result in a more accurate emotion recognition compared to using these variables on their own seems very sensible and has frequently been suggested in the literature as a potential way to improve emotion recognition [16]. However, a few studies explicitly mention that combination of physiological information did not result in reliable improvement (e.g., [17–19]) or only to a modest degree in one of multiple conditions without statistical evidence (e.g., [5]). In this study, the experimental results and the statistical analysis have provided clear evidence for the benefit of multimodal combination for emotion recognition. It could be explained that the emotion state involves multiple processes that are presumably reflected by different types of variables (e.g., cognitive processes by EEG and physical change by peripheral facial expression measures).

In this study, we did find significant improvement for the multimodal fusion detection, compared to the single pattern detection. The reason could be based on the fact that the facial expression detection has a fast and strong but fluctuating response, and the EEG detection had a smooth but stable response over the trial time [20]. Specifically, there is high volatility in real emotion recognition based only on facial expressions because subjects are able to trick the machine as long as they know how to pretend via their facial expressions. In this respect, the drawbacks of facial expression detection can be compensated for by the EEG detection to a very large extent. Thus, the facial expression detection and EEG detection were irreplaceable and complementary to each other, and the multimodal fusion should achieve higher accuracies using both detections than using one of the two detections. This was demonstrated by the data analysis results in Table 2.

While most studies combine information by fusion at the feature level, we thought that fusion of information at the decision level could have contributed to finding a strong reliable advantage of combining information. One the one hand, fusion at this level is difficult to achieve in practice because the feature sets of the various modalities may not be compatible (e.g., brain and peripheral physiological signals in this study) [21]. Most commercial biometric systems do not provide access to the feature sets nor the raw data which they use in their products [22]. On the other hand, the advantage of decision-level fusion is that all knowledge about the different signals can be applied separately [23]. In this study, the facial expression and EEG signals have their own capabilities and limitations as mentioned above, and we can use this information to optimize the detection performance. In the decision-level fusion, it is relatively easy to access and combine the scores generated by neural network and SVM classifiers pertaining to facial expression and EEG modalities. Thus, fusion at the decision level is preferred in this study.

For the decision-level fusion, the classifier selection for facial expression and EEG detections is also important. Several properties have to be taken into consideration, such as the long term variability of facial expression signals and the availability of small data sets of EEG signals. First, the neural network-based methods are found to be particularly promising for facial expression recognition, since the neural networks can easily implement the mapping from the feature space of face images to the facial expression space [24]. Second, a neural network model generally requires a large amount of high-quality data for training. In this study, the EEG signals recorded by a one-electrode mobile device could lack sufficient training data for the neural network-based method. Third, SVM is known to have good generalization properties and to be insensitive to overtraining and to the curse-of-dimensionality, especially in the small data set [25]. It should be noted that SVM classifier was widely used in the EEG-based brain computer interface in practice [26–29]. Furthermore, some modified support vector classification (SVC) methods had the advantage of using a regularization parameter to control the number of support vectors and margin errors. For example, Gu and Sheng developed a modified SVC formulation based on a sum-of-margins strategy to achieve better online accuracy than the existing incremental SVC algorithm [30]. They further proposed a robust SVC method based on lower upper decomposition with partial pivoting, which results in fewer steps and less running time than original one does [31]. Taken together, the neural network classifier was used for the facial expression detection, and the SVM classifier was used for EEG detection in this study.

Two multimodal fusion methods are proposed in this study. For the first fusion method, SVM classified the EEG signal into the four types of emotion, and fusion is performed using a sum rule. For the second fusion method, SVM classified the EEG signal into three intensity levels (weak, moderate, and strong), and fusion is performed using a production rule. It is interesting to note that the second fusion method combining type of emotion and intensity level yields comparable average accuracies with the first fusion method. Indeed, it might very well be what humans do for emotion recognition: for example, an expression of weak happiness is typically answered with neutral, whereas a strong expression of sadness usually evokes fear.

For the results of Experiment 2, average accuracies of 81.25% (online) and 82.75% (offline) were achieved by two fusion methods for four-class emotion recognition. Superior performance was obtained compared to the results in the state-of-the-art results [3, 11, 32]. In fact, the authors of [3] reported an average accuracy of 78.4% by using optical flow to determine the main direction of movement of the muscles. In [11], the IAPS was used as stimuli, and the use of self-assessment labels for arousal assessment yielded accuracies of 55%, 53%, and 54% for EEG and physiological and fused features, respectively. Zheng and his colleges presented an emotion recognition method combining EEG signals and pupillary response collected from eye tracker and achieved average accuracies of 73.59% and 72.98% for three emotion states using feature level fusion strategy and decision-level fusion strategy, respectively.

This study still has open issues that need to be considered in the future. At this present stage, the image data set we obtained is very limited, and the EEG signals used for analysis were recorded from only one electrode. In the future, however, we will collect more image data from more subjects and use a more complicated model to train our data to yield a classifier with better performance. Furthermore, we could consider an EEG device with more electrodes to obtain higher-quality data.

## Figures and Tables

**Table 1:** The production rules of combining the emotion state and intensity level.

*R*_*i*_	*P*	*Q*
*R*_1_	(Happiness, strong)	Happiness
*R*_2_	(Happiness, moderate)	Happiness
*R*_3_	(Happiness, weak)	Neutral
*R*_4_	(Neutral, strong)	Happiness
*R*_5_	(Neutral, moderate)	Neutral
*R*_6_	(Neutral, weak)	Neutral
*R*_7_	(Sadness, strong)	Fear
*R*_8_	(Sadness, moderate)	Sadness
*R*_9_	(Sadness, weak)	Sadness
*R*_10_	(Fear, strong)	Fear
*R*_11_	(Fear, moderate)	Fear
*R*_12_	(Fear, weak)	Sadness

**Table 2:** The accuracies for the detections of face expression, EEG, and two fusion methods.

Subject	Face expression	EEG	The first fusion method (online)	The second fusion method (offline)
1	92.5	67.5	92.5	87.5
2	57.5	70.0	82.5	87.5
3	50.0	72.5	87.5	87.5
4	62.5	75.0	92.5	87.5
5	60.0	60.0	75.0	75.0
6	87.5	75.0	95.0	92.5
7	72.5	72.5	72.5	80.0
8	70.0	70.0	80.0	87.5
9	92.5	60.0	75.0	80.0
10	85.0	62.5	72.5	80.0
11	67.5	72.5	80.0	80.0
12	80.0	75.0	85.0	85.0
13	92.5	57.5	92.5	87.5
14	72.5	55.0	77.5	80.0
15	70.0	52.5	75.0	77.5
16	92.5	62.5	77.5	77.5
17	77.5	57.5	90.0	87.5
18	92.5	80.0	92.5	85.0
19	50.0	62.5	60.0	75.0
20	62.5	77.5	70.0	75.0

*Average*	*74.38 *±* 14.55*	*66.88 *±* 8.19*	*81.25 *±* 9.47*	*82.75 *±* 5.19*
